# Resilience mediates the effect of self-efficacy on symptoms of prenatal anxiety among pregnant women: a nationwide smartphone cross-sectional study in China

**DOI:** 10.1186/s12884-021-03911-5

**Published:** 2021-06-17

**Authors:** Ruqing Ma, Fengzhi Yang, Lijuan Zhang, Kristin K. Sznajder, Changqing Zou, Yajing Jia, Can Cui, Weiyu Zhang, Wenzhu Zhang, Ning Zou, Xiaoshi Yang

**Affiliations:** 1 Department of Social Medicine, School of Public Health, China Medical University, No.77 Puhe Road, Shenyang North New Area, Shenyang, Liaoning Province China; 2 Department of Obstetrics and Gynecology, Shengjing Hospital of China Medical University, Shenyang, Liaoning Province China; 3 Department of Public Health Sciences, College of Medicine, Pennsylvania State University, Hershey, PA USA; 4 Department of Humanities and Social Sciences, China Medical University, Shenyang, Liaoning Province China

**Keywords:** Anxiety, Pregnant women, Resilience, Self-efficacy, Mediation analysis

## Abstract

**Background:**

Prenatal anxiety is one of the most prevalent mental disorders during pregnancy. This study assessed the prevalence of prenatal anxiety and examined whether resilience could play the mediating role in the association between self-efficacy and symptoms of prenatal anxiety among pregnant women in China.

**Methods:**

A nationwide smartphone cross-sectional study was carried out in three cities (Shenyang of Liaoning Province, Zhengzhou of Henan Province and Chongqing Municipality) in China from July 2018 to July 2019. The questionnaire consisted of questions on demographic characteristics, the Generalized Anxiety Disorder Scale (GAD-7), the Chinese version of General Self-efficacy Scale (GSES), and the 14-item Wagnild and Young Resilience Scale (RS-14). A total of 665 pregnant women were recruited in this study. A hierarchical multiple regression model was employed to explore the associate factors and mediators of symptoms of prenatal anxiety. A structural equation model was employed to test the hypothesis that resilience mediates the association between self-efficacy and symptoms of prenatal anxiety.

**Results:**

The prevalence of symptoms of prenatal anxiety was 36.4% in this study. Self-efficacy was negatively correlated with symptoms of prenatal anxiety (*r* = -0.366, *P* < 0.01). Resilience had a significant positive correlation with self-efficacy (*r* = 0.612, *P* < 0.01) and had a negative correlation with symptoms of prenatal anxiety (*r* = -0.427, *P* < 0.01). The hierarchical multiple regression model indicated that self-efficacy and resilience were the main factors associated with symptoms of prenatal anxiety and contributed to 11.9% and 6.3% to the variance of symptoms of prenatal anxiety, respectively. Resilience served as a mediator between self-efficacy and symptoms of prenatal anxiety (a*b = -0.198, Bias-corrected and accelerated bootstrap 95% Confidence interval: -0.270, -0.126).

**Conclusions:**

Self-efficacy was a negative predictor of symptoms of prenatal anxiety among pregnant women. Moreover, resilience mediated the relation between self-efficacy and symptoms of prenatal anxiety among pregnant women in China. It was observed in this study that psychological interventions might be beneficial for pregnant women to relieve symptoms of prenatal anxiety through improved self-efficacy and resilience.

**Supplementary Information:**

The online version contains supplementary material available at 10.1186/s12884-021-03911-5.

## Background

Pregnancy is a major life event among women of childbearing age [1], but can be challenging due to changes in physical and mental function and social roles in both the prenatal and postnatal period. According to a body of previous studies, women are more likely to perceive psychological changes and be susceptible to symptoms of mental disorders including anxiety and depression during pregnancy [2, 3]. It is also widely recognized that women’s mental disorders, particularly for prenatal women, are closely associated with the well-being of both pregnant women and their children [4].

Symptoms of prenatal anxiety are prevalent during the prenatal period. Prenatal anxiety is a state of high tension which a pregnant woman might experience when she is about to give birth due to new expectations, worry, and lack of self-efficacy [5]. A growing number of studies indicate that the prevalence of prenatal anxiety of pregnant women in China is high (7.9 to 68.4%) [6–10]. Moreover, the prevalence of prenatal anxiety in Turkey, South Africa, Pakistan, Australia, Canada, UK and US was reported to be 15.2 ^_^ 49.0% in several studies [10–14]. As a major life event, pregnancy not only results in a series of new challenges for women themselves, but also pushes them to transition into a new role in family and society. Prenatal anxiety may have profound and detrimental effects on maternal and fetal health outcomes. Prenatal anxiety has been found to be associated with an elevated odds of postnatal mental disorders, obstetric diseases, the decline of well-being, fetal abnormalities, infants' temperamental negative affectivity (NA), stillbirth, and poor children's growth and development [7, 15–19]. However, the mental health care service especially for pregnant women remains deficient and unavailable. Given the limited health services provided to pregnant women, the need for interventions and treatment for pregnant women has become a serious public health issue.

Self-efficacy, a widely-acknowledged positive psychological resource, has a significant meaning to women when they experience the period to transit into motherhood [20]. As explained by Bandura’s cognitive social theory [21, 22], human behavior is self-regulated, namely, their behaviors are triggered by their own self-efficacy beliefs [22]. Self-efficacy was defined as “people’s judgment of their capabilities to organize and execute courses of action required to attain designated types of performance” [23]. Numerous previous researches have indicated that perceived self-efficacy is a key construct and the most proximal determinant of behavior [20, 24]. Self-efficacy can influence maternal physical and psychological states such as anxiety and depression [24, 25]. Self-efficacy can also help individuals cope with stress rationally and positively [6, 23]. Moreover, self-efficacy appears to be particularly important to overcoming barriers that might hinder improved pregnancy-related health outcomes [26], such as prenatal anxiety [7].

Resilience, another positive psychological resource to prevent mental disorders, is a dynamic process that enables individuals at any stage of life to cope with adversity, bounce back after hardship, handle unpleasant feelings, and adapt to change [25, 27–29]. Resilience can also help pregnant women prevent or minimize adverse damage, and recover from or overcome the difficulties of challenging environments. In addition, women with high levels of resilience could positively adapt to the process of pregnancy and confront adversity in healthy ways. Prior research has revealed that resilience is crucial for pregnant women to maintain mental health [27]. Some recent researches have also found that resilience serves as a mediator between mindfulness and anxiety among pregnant women [18, 30].

The aspects of resilience as a process of self-adjustment may not only prevent symptoms of anxiety [31], but also may mediate other adverse impacts of anxiety such as preterm birth [32–34]. Some studies support the hypothesis that resilience is positively correlated with self-efficacy [35, 36]. One recent research study has shown that resilience could mediate the effect of self-efficacy on grit [35]. Additionally, a Chinese study on family members of patients in intensive care found that resilience mediated the association between self-efficacy and symptoms of anxiety [37]. Furthermore, for Chinese pregnant women, new research has shown that resilience could reduce symptoms of prenatal anxiety and play a mediating role in the relationship between maternal stress and symptoms of prenatal anxiety [18]. However, the researched which measures resilience among Chinese pregnant women and related impacts on symptoms of prenatal anxiety are still scarce. The potential relationship of self-efficacy, resilience, and symptoms of prenatal anxiety has not been examined among pregnant women in previous studies. Therefore, this study hypothesized that 1) self-efficacy and resilience are associated with symptoms of prenatal anxiety among pregnant women; and 2) self-efficacy is associated with resilience; and 3) resilience mediates the association between self-efficacy and symptoms of prenatal anxiety among pregnant women.

## Methods

### Study design and sample

A cross-sectional nationwide study designed with a two phase multi-stage sampling was conducted with smartphone questionnaire in three cities from two provinces and one municipality (Shenyang of Liaoning Province, Zhengzhou of Henan Province and Chongqing Municipality) in China from July 2018 to July 2019. In the first-stage, a tertiary hospital was selected in each city by random sampling, including the Second Affiliated Hospital of China Medical University, Zhengzhou Maternal and Child Health Hospital and Chongqing Bishan district people's hospital. In the second-stage, approximately 30% of the pregnant women who underwent routine prenatal examinations at the obstetrics clinics of these three hospitals were selected by probability sampling as the participants of our study. The inclusion criteria comprised of: 1) pregnant women aged over 18, 2) able to comprehend the smartphone questionnaire and willingness to take part in the study, and 3) legally married in China (in the context of traditional Chinese culture and ethics, legal marriage is the standard for citizens, and legal marriage ensures that women can legally give birth). The exclusion criteria were 1) pregnant woman who were clinically diagnosed with mental disorders, such as schizophrenia, severe depression and anxiety, mania, and bipolar affective disorder, etc., 2) abnormal pregnancy such as fetal malformation, 3) previous mental illness and cognitive dysfunction.

The recruited pregnant women were well informed of the purposes and details of the survey in advance. Then, electronic consent forms were signed by the participants and the smartphone electronic questionnaires were collected. A face-to-face interview (taking 15–20 min to complete) was conducted by trained surveyors. All of the processes of this survey followed the ethical standards of the committee on human experimentation at the China Medical University (Shenyang, China) and were approved by that committee. Among the total 800 women identified, 665 women completed the questionnaire, leading to a valid response rate of 83.1% (See Supplementary File 1, 2, Additional Files 1, 2).

### Demographic characteristics

Demographic information regarding age, education, monthly income, gravidity, gestational week, and number of chronic disease were collected in this survey. Education level was grouped as junior college or lower, and college or higher. Gravidity was divided into once and twice or more. A variable capturing chronic disease was dichotomized as at least one chronic disease and no chronic disease.

### Measurement of prenatal anxiety symptoms

The Generalized Anxiety Disorder Scale (GAD-7) was applied to evaluate the symptom of anxiety [38]. The scale consists of 7 items and the scores for each item range from at 0 (completely not/ no difficulty) to 3 (almost daily/ extremely difficult). The sum score of GAD-7 is with the range of 0 ~ 21, of which 5 was used as the cut-off value of the scale to define anxiety [39, 40]. A higher score indicated a higher level of anxiety. The Cronbach’s alpha for GAD-7 in this study was 0.937.

### Measurement of self-efficacy

Self-efficacy was assessed by the Chinese version of General Self-efficacy Scale (GSES), developed by Schwarzer [41]. This scale has good reliability and validity when employed in Chinese populations [42]. The scale included 10 items with a 4-point Likert scale. A higher overall score indicated a higher degree of self-efficacy. In the present study, the Cronbach’s alpha for GSES in this study was 0.953.

### Measurement of resilience

Resilience was evaluated with the 14-item Wagnild and Young Resilience Scale (RS-14), using is a 7-point Likert scale and appropriate in various populations [43]. Each item was ranging from 1 (strongly disagree) to 7 (strongly agree). A higher total score indicates higher levels of resilience. The Cronbach’s alpha for RS-14 in this study was 0.957.

### Statistical analysis

All of the statistical analyses were carried out with the Statistical Package for Social Sciences (SPSS) for Windows version 17.0. A two-sided P < 0.05 were considered to be significant. T-tests and Analysis of Variances (ANOVAs) were applied to compare differences in symptoms of prenatal anxiety among categorical groups. The Spearman correlation was employed to explore the correlations of self-efficacy, resilience, and symptoms of prenatal anxiety. Hierarchical Multiple Regression (HMR) analysis was conducted to explore associate factors and mediators of prenatal anxiety symptoms. The GAD-7 score was used as the dependent variable, the independent variables were entered as follows: In step 1, *demographic characteristics* of the pregnant women; in step 2, *self-efficacy*; in step 3, *resilience*. The contributions to the HMR models were tested with blocks of independent variables entered in later stages [44]. Based on the Sobel test on mediation [45], variables were entered in the order by which their incremental contributions were examined. The mediating role of resilience in the association between self-efficacy and symptoms of prenatal anxiety was tested with a Structural Equation Model (SEM), with the analysis of Amos 17.0. The dependent variable was symptoms of prenatal anxiety, the independent variable was self-efficacy and the mediator was resilience; and all variables were consist with the SEM criteria (χ2/df less than 5, Goodness of fit index (GFI) high than 0.90, Comparative fit index (CFI) high than 0.90, Root mean square error of approximation (RMSEA) high than 0.08, and Tucker-Lewis index (TLI) high than 0.90). The mediating effect (a*b product) of resilience in the association between self-efficacy and symptoms of prenatal anxiety was analyzed with bootstrapping, with an estimate of 5000 samples. The bias-corrected and accelerated 95% confidence interval ( Bias-corrected and accelerated bootstrap (BCa) 95% Confidence interval (CI)) for a*b estimates were tested by SEM. All study variables were standardized prior to analysis to account for differences in scale scores.

## Results

### Participant characteristics

Basic characteristics of the pregnant women and the distribution of prenatal anxiety symptoms are shown in Table 1. The mean prenatal anxiety symptoms score in the study population was 3.91(Standard Deviation (SD) = 4.27). The prevalence of prenatal anxiety symptoms was 36.4% in this study. Among the 665 pregnant women, their age ranged from 19 to 42 years (Mean (M) = 29.91, SD = 4.031). In this study, 41.5% of the pregnant women had a monthly income level more than ¥5,000 (US $772.38). About 7.7% of pregnant women suffered from chronic diseases such as chronic hypertension and diabetes. Pregnant women with a monthly income more than ¥5,000 (US $772.38) reported a lower score of prenatal anxiety (*P* < 0.05) than those with a monthly income of less than ¥3,000 (US $463.43). Pregnant women who experienced chronic diseases reported a significantly higher levels of symptoms of prenatal anxiety (*P* < 0.01) than those without a chronic disease. Meanwhile, age, education, gravidity, and gestational week were not statistically association with symptoms of prenatal anxiety (*P* > 0.05). Symptoms of prenatal anxiety tended to be worse among pregnant women with a lower monthly income level and among women with a chronic disease.

**Table 1:** Demographic characteristics and differences in symptoms of prenatal anxiety (*N*^c^ = 665)

Variables	Number (n)	Percent (%)	symptoms of prenatal anxiety (Mean ± SD)
**Age (years)**			
< 30	322	48.4	4.158 ± 4.515
30–34	246	37.0	3.683 ± 3.982
≥ 35	97	14.6	3.691 ± 4.111
**Education**			
Junior college and below	331	49.8	4.094 ± 4.283
Undergraduate and above	334	50.2	3.737 ± 4.250
**Monthly income (¥)**^**d**^			
≤ 3000	102	15.3	4.755 ± 4.406
3001–5000	287	43.2	3.927 ± 4.122
> 5000	276	41.5	3.591 ± 4.335*^a^
**Gravidity**			
Once	365	54.9	3.937 ± 4.234
Twice or more	300	45.1	3.887 ± 4.314
**Gestational week (weeks)**			
≤ 13	86	12.9	4.034 ± 4.436
14–27	173	26.0	4.376 ± 3.848
≥ 28	406	61.1	3.692 ± 4.392
**Chronic diseases**			
No	614	92.3	3.770 ± 4.140
Yes	51	7.7	5.647 ± 5.329**^b^

Abbreviations:

^a^* *P* < 0.05

^b^** *P* < 0.01 (two-tailed)

^c^N: Number

^d^1¥ ≈ US $0.154

### Correlations among continuous variables

Correlations among the continuous variables are illustrated in Table 2. Age was positively correlated with self-efficacy and resilience significantly. Symptoms of prenatal anxiety was significantly negatively correlated with self-efficacy (*r* =  − 0.366, *p* < 0.01) and resilience (*r* =  − 0.427, *p* < 0.01). Furthermore, self-efficacy had a positive correlation with resilience (*r* = 0.612, *p* < 0.01).

**Table 2:** Correlations between symptoms of prenatal anxiety and related factors

Variables	1	2	3
1. Symptoms of prenatal anxiety	1		
2. Self-efficacy	-0.366**^a^	1	
3. Resilience	-0.427**	0.612**	1

Abbreviations:

^a^** *P* < 0.01 (two-tailed)

### Regression analysis of self-efficacy, resilience, and symptoms of prenatal anxiety

The final HMR analyses to explore the mediating effect of resilience as presented in Table 3. Each block of the independent variables contributed to the variance in the HMR model of symptoms of prenatal anxiety. A total of 21.2% of variance was interpreted by the final HMR model for symptoms of prenatal anxiety. R^2^ change hints that self-efficacy contributed most to the variance of symptoms of prenatal anxiety (11.9% of variance, β =  − 0.354, *P* < 0.001), while resilience contributed second-most to the variance of symptoms of prenatal anxiety (6.3% of variance, β =  − 0.320, *P* < 0.001). In addition, based on the Sobel test, resilience partially mediated self-efficacy on symptoms of prenatal anxiety (standardized regression coefficient (β) reduced from 0.354 to 0.164).

**Table 3:** The hierarchical multiple regression models of symptoms of prenatal anxiety

Variables	Model 1 b(B)^b^	Model 2 b(B)	Model 3 b(B)
**BLOCK1 Demographic characteristics**			
Age	-0.069	-0.032	-0.028
Monthly income (¥) ^e^			
3001-5000vs. ≤ 3000	-0.077	-0.042	-0.015
> 5000vs. ≤ 3000	-0.105	-0.029	0.018
Education	-0.020	-0.017	-0.023
Chronic diseases	-0.126**^a^	-0.110**	-0.100**
Gravidity	-0.034	-0.019	-0.022
Gestational week	-0.022	-0.023	-0.013
**BLOCK 2 Self-efficacy**		-0.354**	-0.164**
**BLOCK 3 Resilience**			-0.320**
**R**^**2 c**^	0.030	0.149	0.212
**△R**^**2 d**^	0.039	0.119	0.063

Abbreviations: ^a^** *P* < 0.01 (two-tailed)

^b^b(B) = Beta (Standardized regression coefficient)

^c^R^2^: square of ratio

^d^△R^2^: the difference of the square of ratio

^e^1¥ ≈ US $0.154

### Resilience mediated self-efficacy on symptoms of prenatal anxiety

**Fig. 1:**
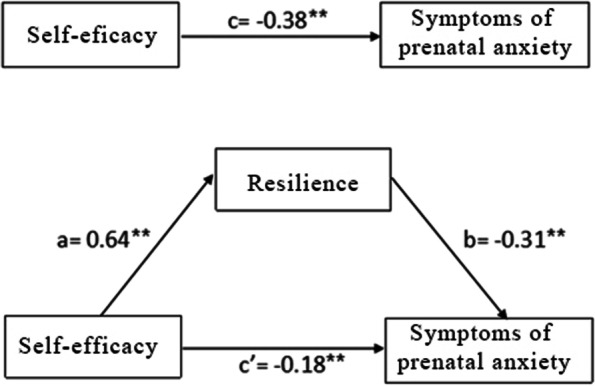
The Amos model of the mediating role of resilience on the association between self-efficacy and symptoms of prenatal anxiety. c: associations of self-efficacy with symptoms of prenatal anxiety; a: associations of self-efficacy with resilience; b: association between self-efficacy and symptoms of prenatal anxiety after controlling for the predictor variables; c’: associations of self-efficacy with symptoms of prenatal anxiety after adding resilience as a mediator

The direct pathways of self-efficacy with symptoms of prenatal anxiety through resilience are shown in Fig. 1. The SEM model presented the significant negative associations of self-efficacy with symptoms of prenatal anxiety (c =  − 2.85, *P* < 0.01) (Fig. 1), which was with a good model fit according to the SEM criteria ( X^2^/df < 5, *p* < 0.01, GFI = 0.957, AGFI = 0.930, CFI = 0.980, TLI = 0.974, and RMSEA = 0.066).

The mediating effect of resilience on the association between self-efficacy with symptoms of prenatal anxiety is presented in Fig. 1. The path coefficient of self-efficacy with symptoms of prenatal anxiety reduced significantly when resilience was entered as a mediator. The bias-corrected and accelerated bootstrap test indicated that resilience significantly mediated the association of self-efficacy and symptoms of prenatal anxiety (a*b =  − 0.198, BCa 95% CI: -0.270 ~ -0.126). These models verified that resilience as the mediator in the relationship between self-efficacy and symptoms of prenatal anxiety, which is also a good model fit according to the SEM criteria ( X^2^/df < 5, RMSEMA = 0.069, CFI = 0.975, GFI = 0.946, AGFI = 0.918, and TLI = 0.968) (Fig. 1). Thus, self-efficacy directly impacts symptoms of prenatal anxiety, and also influences symptoms of prenatal anxiety indirectly through the resilience path.

## Discussion

Deficient researched have investigated the relationship between positive psychological resources and symptoms of prenatal anxiety among pregnant women, this study explored resilience’s mediating effects on the association between self-efficacy and symptoms of prenatal anxiety of Chinese pregnant women. In the present study, 36.4% of pregnant women reported prenatal anxiety, which is higher than the results from previous studies in China: 15.04% in Chongqing; 11.1% in Shanghai; and 7.98% in Guangxi [7, 8, 18], but lower than the prevalence (68.4%) in a study conducted in Beijing [9]. Meanwhile, in previous studies from Turkey, South Africa and Pakistan, the prevalence of prenatal anxiety has been reported to be 15.9, 23 and 49% respectively [10–14]. Moreover, this study observed that the prevalence of anxiety was almost twice that reported in a systematic review on prenatal anxiety in 34 countries (18.2%) [13]. As has been shown in the literature, Asians tend to be more conservative than Westerners on some sensitive issues because of differences in population, culture, customs, and norms [46, 47]. Meanwhile, the western concept of perinatal care, which is different from the traditional oriental health care advocated by parents, were increasingly accepted by women in China. Culture clashes may exacerbate prenatal stress and prenatal disorders such as anxiety [48]. Further, “Western” measures and standards were commonly applied by researchers in their studies on mental disorders, and cultural differences may lead Asian populations to ignore specific symptoms in China [49]. Above conditions may explain the higher rate of prenatal anxiety among Chinese women.

Results from this research support the hypothesis that symptoms of prenatal anxiety are negatively associated with self-efficacy. Furthermore, resilience mediates in promoting the effects of self-efficacy on relieving symptoms of prenatal anxiety, which may throw lights on the idea that the Chinese pregnant women when faced with anxiety symptoms could optimize the attenuating effects of self-efficacy through increasing resilience in order to reduce symptoms of prenatal anxiety.

In this study, chronic disease was found to be a significant associate factor of symptoms of prenatal anxiety. Pregnant individuals suffering from chronic disease usually encounter numerous additional negative impacts on their own mental health. Chronic hypertension, for example, affects up to 5% of pregnancies which are susceptible to increased adverse maternal and neonatal outcomes [50]. Pregnancy stress can cause symptoms of prenatal anxiety [7, 8]. This finding is corroborated by other research showing women who experienced chronic diseases tended to have higher risks of symptoms of prenatal anxiety [7].

Notably, this study found that self-efficacy contributed the most to symptoms of prenatal anxiety and had a negative association with symptoms of prenatal anxiety which verified hypothesis 1. Pregnant women with higher self-efficacy reported lower anxiety symptoms, which was inconsistent with previous studies [23, 51]. Pregnant women with higher self-efficacy may be better at coping with anxiety symptoms compared with those with lower self-efficacy. Based on Bandura’s theory [21], anxiety symptoms could be regulated and triggered by higher perceived levels of self-efficacy. In our study, self-efficacy had strong direct effects on symptoms of prenatal anxiety, accounting for 11.9% of the total variance. This implies that high levels of self-efficacy could be utilized to contend with symptoms of prenatal anxiety. Moreover, according to the physiological responses of self-efficacy [24], a positive belief about one’s abilities to succeed at challenging tasks are established [51]. Thus, self-efficacy could enhance pregnant women to adjust pregnancy and prevent from prenatal anxiety. During pregnancy, pregnant women face changes in their physiology and social roles, as well as physical discomforts. Pregnant women with a high sense of self-efficacy may be better able to prepare for childbirth, find new interests, invest in new changes, adapt to various physical and psychological discomforts and environmental changes, which could enhance resilience [21–23]. Pregnant women with high self-efficacy contributing to effective and confident coping mechanisms in stressful situations may have stronger capabilities to overcome stress during pregnancy and be more resilient [52].

Remarkably, it is observed in this study that resilience mediates the impact of self-efficacy on symptoms of prenatal anxiety. Similarly, recent literature has indicates the mediating effects of resilience in the relationship between maternal stress and symptoms of prenatal anxiety of pregnant women was exist [14]. Previous research on resilience suggests that higher resilience is linked with better mental health and may promote the positive effect of self-efficacy on symptoms of prenatal anxiety [18, 53].

Resilience, which can prevent the symptoms of prenatal anxiety, may guard against negative health outcomes including low birth weight (LBW) and preterm birth [53, 54]. During the prenatal period, pregnant women may experience symptoms of prenatal anxiety as a result of an insufficient understanding of the delivery process and concerns about childbirth [55]. Stress in early pregnancy may worsen the mental health of pregnant women compared with stress at other times during pregnancy [56]. Negative cognitive assessment of childbirth is associated with a lower sense of childbirth self-efficacy and resilience, leading pregnant women to respond to childbirth with fear and anxiety [55]. Besides, resilience can be employed as a positive capability to enhance self-efficacy in the face of adversity during pregnancy [35, 36]. In particular, Bandura indicates that resilience has demonstrated a positive association with self-efficacy [23, 36], and may enhance maternal mental health during pregnancy [57, 58], thus resulting in protection from symptoms of prenatal anxiety. Based on Bandura’s theory of social cognition, pregnant woman's beliefs and motivations are inspired by high resilience and often guide their behaviors to face pregnancy events positively. In turn, these positive beliefs and motivations influence and ultimately determine the content and form of thinking about changes in pregnancy, which can reduce prenatal anxiety symptoms [21–23]. Enhanced resilience may boost the attenuating effect of symptoms of prenatal anxiety among pregnant women to regulate their emotions and thoughts in the face of pregnancy complications [58]. At this level, pregnant women with resilience could control negative emotions and strive for positive mental well-being. Subsequently, pregnant women may be encouraged to adapt to new challenges with new approaches during periods with more stress and vulnerability, such as pregnancy, and more actively handle the symptoms of prenatal anxiety [59]. Meanwhile, various studies have found that self-efficacy could be affected by personal psychological resources, such as resilience, in regards to symptoms of prenatal anxiety during pregnancy [22, 23].

In conclusion, resilience is an important element which should be addressed in future intervention to alleviate symptoms of prenatal anxiety. The result of this study contributes to the enhancement of the comprehension that the pregnant women need to be aware of their mental health and develop positive resources, such as positive self-efficacy and resilience, to help them combat the effects of anxiety disorders during pregnancy. Thus, our results have practical significance for Chinese pregnant women to maintain mental well-being. Based on our study results, it is advisable that interventions are developed to enhance resilience and self-efficacy and prevent the symptoms of prenatal anxiety among pregnant women.

### Strengths and limitations

Several strengths are presented in this study. First, the large sample size and sampling procedures, allows for the generalizability of our research conclusions to the locations where data were collected. Second, face-to-face interviews with smartphones resulted a high response rate, ensuring the accuracy of the information. Third, the SEM model illustrates the mediating effect of resilience.

Meanwhile, this study had also limitations. Further studies are needed due to the limits of generalization of the cross-sectional design of this study. Although geographically diverse, this study was only carried out in 3 cities in China, which may also limits the generalizability of the results among pregnant women to other populations. Additionally, the R2 in the HMR model was relatively small, which may not be strong enough to explain the mediating effect.

## Conclusions

The finding of this study revealed that the prevalence of symptoms of prenatal anxiety in China was high (36.4%). Self-efficacy had a negative association with symptoms of prenatal anxiety. Resilience mediated the effect of self-efficacy on symptoms of prenatal anxiety among Chinese pregnant women. Thus, improving self-efficacy and resilience may be beneficial in the prevention of prenatal anxiety symptoms.

## Supplementary Information

**Additional file 1: Supplementary file 1.** English version of questionnaire in this survey, including informed consent, demographic characteristics, GAD-7, GSE and RS-14.

**Additional file 2: Supplementary file 2.** Interview guide for the questionnaire in this survey.

## Data Availability

The datasets used and/or analysed during the current study available from the corresponding author on reasonable request.

## Abbreviations

ANOVAs: Analysis of Variances
BCa: Bias-corrected and accelerated bootstrap
CFI: Comparative fit index
CI: Confidence interval
GAD-7: Generalized Anxiety Disorder Scale
GFI: Goodness of fit index
GSES: General Self-efficacy Scale
HMR: Hierarchical multiple regression
M: Mean
NA: Negative affectivity
OR: Odds ratio
RMB: RenMinBi
RMSEA: Root mean square error of approximation
RS-14: Resilience Scale
SD: Standard Deviation
SEM: Structural equation model
TLI: Tucker-Lewis index
