# Supplementary material for: Resilience mediates the effect of self-efficacy on symptoms of prenatal anxiety among pregnant women: a nationwide smartphone cross-sectional study in China
Source: BMC Pregnancy Childbirth. 2021 Jun 17;21:430. doi: 10.1186/s12884-021-03911-5 (PMC8212491; doi:10.1186/s12884-021-03911-5)
Supplement: Supplementary file 1 — Additional file 1: Supplementary file 1. English version of questionnaire in this survey, including informed consent, demographic characteristics, GAD-7, GSE and RS-14. [file 12884_2021_3911_MOESM1_ESM.docx]

**Additional file 1:** **Supplementary file 1:** English version of questionnaire in this survey, including informed consent, demographic characteristics, GAD-7, GSE and RS-14

**The title of the manuscript: Resilience mediates the effect of self-efficacy on symptoms of prenatal anxiety among pregnant women: A nationwide smartphone cross-sectional study in China**

**The author list:** Ruqing Ma^1#^, Fengzhi Yang^1#^,Lijuan Zhang^2^, Kristin K. Sznajder^3^, Changqing Zou^4^, Yajing Jia^1^, Can Cui^1^, Weiyu Zhang^1^, Wenzhu Zhang^2^, Ning Zou^2^, Xiaoshi Yang^1,^*

**#Co-first authors**: Ruqing Ma and Fengzhi Yang were the Co-first authors. These authors contributed to the work equally and should be regarded as co-first authors.

***Corresponding author**

**Xiaoshi Yang**, PhD, FAX:862431939406, **+**86-18900910796, [xsyang@cmu.edu.cn](mailto:xsyang@cmu.edu.cn), Department of Social Medicine, School of public health, China medical university, No.77 Puhe Road, Shenyang North New Area, Shenyang, Liaoning Province, 110013, P.R. China.

^1^Department of Social Medicine, School of Public Health, China Medical University, No.77 Puhe Road, Shenyang North New Area, Shenyang, Liaoning Province, 110013, P.R. China.

^2^Department of Obstetrics and Gynecology, Shengjing Hospital of China Medical University, No.36 Sanhao Street, Shenyang, Shenyang, Liaoning Province, 110013, P.R. China.

^3^Department of Public Health Sciences, College of Medicine, Pennsylvania State University, 90 Hope Drive, Suite 2200, Hershey, PA 17033. USA.

^4^Department of Humanities and Social Sciences, China Medical University, No.77 Puhe Road, Shenyang North New Area, Shenyang, Liaoning Province, 110013, P.R. China.

**English version of questionnaire in this survey, including informed consent, demographic characteristics, GAD-7, GSE and RS-14**

The assessment of symptoms of prenatal anxiety among pregnant women

Hello!

Informed consent

We will invite you to participate in a research survey organized by China Medical University. The research will help to provide high-quality health care services and prevent the prevalence of psychological disorders in the process of pregnancy.

This study will be conducted via a smartphone questionnaire on the WeChat platform. If you are willing to participate in this study, you will be asked to answer the smartphone questionnaire, together with our staff, and it will take you about 15-20 minutes. All the data you provide will only be used for research purposes, only researchers can access it, and will be destroyed three years after the end of the research. Thus, all your information is absolutely confidential.

Whether you participate in this study is completely voluntary and will not bring you any adverse effects. If you have any questions about this survey, please contact the researchers.

**Agree to participate in research**

I have read the informed consent form and understood the content of the consent form. Now I agree to participate in this project, and I am willing to accept the services provided by the project and fulfill my obligations.

**The characteristics of pregnant women**

Please tick “√” or fill in the “□” before the appropriate option

**A1** What is your age? □□

**A2** Your education level: □Elementary school and below □Junior high school □Senior high school or technical secondary school □Junior college □University and above

**A3** The average monthly total income per person in your family is:

□Less than 2000 yuan □2001-3000 yuan □3001-5000 yuan □5001-10,000 yuan □10001-20,000 yuan □over 20,000 yuan

**A4** How many weeks are you pregnant: ______

**A5** How many times have you been pregnant: □1 □2 □3 □>3

**A6** Do you have any of the following chronic diseases (such as hypertension, diabetes, etc.) or family genetic history (multiple choices available):

□None □Hypertension □Coronary heart disease □Stroke □Diabetes □Chronic obstructive pulmonary disease □Malignant tumors □Mental disorders and psychosis □Chronic nephritis □Asthma □Other chronic diseases □Family genetic history

**A.The 7-item Generalized Anxiety Disorder Scale (GAD-7)**

| During the last 2 weeks, how often have you been troubled by any of the following issues? | Not at all | Several days | More than half the days | Nearly every day |
| --- | --- | --- | --- | --- |
| 1 Feeling nervous, anxious or on edge | 0 | 1 | 2 | 3 |
| 2 Not being able to stop or control worrying | 0 | 1 | 2 | 3 |
| 3 ... | 0 | 1 | 2 | 3 |
| 4 ... | 0 | 1 | 2 | 3 |
| 5 ... | 0 | 1 | 2 | 3 |
| 6 ... | 0 | 1 | 2 | 3 |
| 7 Feeling afraid as if something awful might happen | 0 | 1 | 2 | 3 |

**B.The General Self-Efficacy Scale (GSES)**

| items | Not at all true | Hardly true | Moderately true | Exactly  true |
| --- | --- | --- | --- | --- |
| 1. I can manage to solve difficult problems if I try hard enough | 1 | 2 | 3 | 4 |
| 2. If someone opposes me, I can find the means and ways  to get what I want | 1 | 2 | 3 | 4 |
| 3.... | 1 | 2 | 3 | 4 |
| 4. ... | 1 | 2 | 3 | 4 |
| 5. ... | 1 | 2 | 3 | 4 |
| 6. ... | 1 | 2 | 3 | 4 |
| 7. ... | 1 | 2 | 3 | 4 |
| 8. ... | 1 | 2 | 3 | 4 |
| 9. ... | 1 | 2 | 3 | 4 |
| 10. I can usually handle whatever comes my way | 1 | 2 | 3 | 4 |

**C.The 14-item resilience scale, RS-14**

| items | Strongly disagree | Disagree | Slightly disagree | neutral | Slightly agree | Agree | Strongly agree |
| --- | --- | --- | --- | --- | --- | --- | --- |
| 1. I usually manage one way or another | 1 | 2 | 3 | 4 | 5 | 6 | 7 |
| 2. I feel proud that I have accomplished things in life | 1 | 2 | 3 | 4 | 5 | 6 | 7 |
| 3. ... | 1 | 2 | 3 | 4 | 5 | 6 | 7 |
| 4. ... | 1 | 2 | 3 | 4 | 5 | 6 | 7 |
| 5. ... | 1 | 2 | 3 | 4 | 5 | 6 | 7 |
| 6. ... | 1 | 2 | 3 | 4 | 5 | 6 | 7 |
| 7. ... | 1 | 2 | 3 | 4 | 5 | 6 | 7 |
| 8. ... | 1 | 2 | 3 | 4 | 5 | 6 | 7 |
| 9. ... | 1 | 2 | 3 | 4 | 5 | 6 | 7 |
| 10. ... | 1 | 2 | 3 | 4 | 5 | 6 | 7 |
| 11. ... | 1 | 2 | 3 | 4 | 5 | 6 | 7 |
| 12. ... | 1 | 2 | 3 | 4 | 5 | 6 | 7 |
| 13. ... | 1 | 2 | 3 | 4 | 5 | 6 | 7 |
| 14. When I'm in a difficult situation, I can usually find my way out of it | 1 | 2 | 3 | 4 | 5 | 6 | 7 |

Thank you very much for participating in our research!
