# Supplementary material for: Resilience mediates the effect of self-efficacy on symptoms of prenatal anxiety among pregnant women: a nationwide smartphone cross-sectional study in China
Source: BMC Pregnancy Childbirth. 2021 Jun 17;21:430. doi: 10.1186/s12884-021-03911-5 (PMC8212491; doi:10.1186/s12884-021-03911-5)
Supplement: Supplementary file 2 — Additional file 2: Supplementary file 2. Interview guide for the questionnaire in this survey. [file 12884_2021_3911_MOESM2_ESM.docx]

**Additional file 2: Supplementary file 2:** Interview guide for the questionnaire in this survey

**The title of the manuscript: Resilience mediates the effect of self-efficacy on symptoms of prenatal anxiety among pregnant women: A nationwide smartphone cross-sectional study in China**

**The author list:** Ruqing Ma^1#^, Fengzhi Yang^1#^, Lijuan Zhang^2^, Kristin K. Sznajder^3^, Changqing Zou^4^, Yajing Jia^1^, Can Cui^1^, Weiyu Zhang^1^, Wenzhu Zhang^2^, Ning Zou^2^, Xiaoshi Yang^1,^*

**#Co-first authors:** Ruqing Ma and Fengzhi Yang were the Co-first authors. These authors contributed to the work equally and should be regarded as co-first authors.

***Corresponding author**

**Xiaoshi Yang**, PhD, FAX:862431939406, **+**86-18900910796, [xsyang@cmu.edu.cn](mailto:xsyang@cmu.edu.cn), Department of Social Medicine, School of public health, China medical university, No.77 Puhe Road, Shenyang North New Area, Shenyang, Liaoning Province, 110013, P.R. China.

^1^Department of Social Medicine, School of Public Health, China Medical University, No.77 Puhe Road, Shenyang North New Area, Shenyang, Liaoning Province, 110013, P.R. China.

^2^Department of Obstetrics and Gynecology, Shengjing Hospital of China Medical University, No.36 Sanhao Street, Shenyang, Shenyang, Liaoning Province, 110013, P.R. China.

^3^Department of Public Health Sciences, College of Medicine, Pennsylvania State University, 90 Hope Drive, Suite 2200, Hershey, PA 17033. USA.

^4^Department of Humanities and Social Sciences, China Medical University, No.77 Puhe Road, Shenyang North New Area, Shenyang, Liaoning Province, 110013, P.R. China.

**Interview guide for the questionnaire in this survey**

Interview Guide

Firstly, the researcher explained the purpose of the interview to the selected interviewees and the confidentiality of the survey data. Secondly, the interviewees were asked whether they would like to participate. If willing, the informed consent will be signed, and the following face to face interview will be conducted until the end of the questionnaire interview. If the interviewee is unwilling to participate in the interview at the beginning or wants to terminate the interview during the interview, the interview survey will be terminated and over. After the survey is ended or finished, the next interviewee interview survey will be conducted.

Interviewer

The first question for each item should be asked exactly as written. Often this question will elicit enough information about the severity and frequency of a event or symptom for you to rate the item with confidence. Follow-up questions are pro-vided, however, for use when further exploration or additional clarification of events or symptoms is necessary. The specified questions should be asked until you have enough information to rate the item confidently. In some cases, you may also have to add your own follow-up questions to obtain necessary information.

For objective questions(A1-A6), the interviewer asks the participants according to the questions and fills in the answers.

**Notes:** Question A3, the frequency is per person per month about household income.

Question A4, the week time of pregnant is filled in according to the hospital's inspection report.

Question A5, the number of pregnancies includes the number of this pregnancy and previous pregnancies and abortions.

**A1** What is your age? □□

**A2** Your education level: □Elementary school and below □Junior high school □Senior high school or technical secondary school □Junior college □University and above

**A3** What is the average monthly total income per person in your family?

□Less than 2000 yuan □2001-3000 yuan □3001-5000 yuan □5001-10,000 yuan □10001-20,000 yuan □over 20,000 yuan

**A4** How many weeks are you pregnant: ______

**A5** How many times have you been pregnant: □1 □2 □3 □>3

**A6** Do you have any of the following chronic diseases (such as hypertension, diabetes, etc.) or family genetic history (multiple choices available):

□None □Hypertension □Coronary heart disease □Stroke □Diabetes □Chronic obstructive pulmonary disease □Malignant tumors □Mental disorders and psychosis □Chronic nephritis □Asthma □Other chronic diseases □Family genetic history

**A. The 7-item Generalized Anxiety Disorder Scale (GAD-7)**

**Overview:**

I'd like to ask you some questions about the past 2 weeks. How often have you been troubled by any of the following issues? Or, how have you been feeling since last 2 weeks (day of week)?

If there are these phenomena, please ask: How much have these things been bothering you this past week? (How bad have they gotten? How much of the time, or how often, have you had them? How often has that happened)

**Notes:**

*Time period*. During the last 2 weeks.

If there is no such emotion or event, the answer is 0-Not at all. If there is such emotion or event, the participant continue to answer how many days have been this emotion in the past 2 weeks.

| During the last 2 weeks, how often have you been troubled by any of the following issues? |  |
| --- | --- |
| 1 Feeling nervous, anxious or on edge  (In this past 2 weeks, have you felt nervous, anxious or upset, maybe due to pregnancy or work or life events, like (Childbirth anxiety, self anxiety, fetal anxiety) ?) | 0-Not at all, No such emotion happened  1-Several days, One day or one time and half of the time in the past two weeks  2-More than half the days  3-Nearly every day, Frequently |
| 2 Not being able to stop or control worrying (In this past 2 weeks, have you been unable to stop or control the worry, like worrying about the health of yourself and the baby, or whether the birth can be successfully, or some work and life problems, maybe due to pregnancy or work or life events? ) |  |
| 3 ... |  |
| 4 ... |  |
| 5 ... |  |
| 6 ... |  |
| 7 Feeling afraid as if something awful might happen (In this past 2 weeks, have you been feeling as if something terrible or bad will happen and be afraid, like fear of unknown childbirth?) |  |

1. **The General Self-Efficacy Scale (GSES)**

**Overview:**

During your pregnancy and previous life, you must have encountered many difficulties. Here are some typical situations. Please measure as objectively as possible, in each situation, to what extent can you have such behaviors or ideas? Here "1" means Exactly true (totally agree), and "4" means Not at all true (totally disagree).

Please answer according to your actual situation.

| items |  |
| --- | --- |
| 1. I can manage to solve difficult problems if I try hard enough (When encountering a difficult and troublesome situation, as long as you work hard to find a way to change a little bit to deal with the problem, you can finally solve the difficult problems, including difficulties caused by pregnancy.) | 1-Not at all true, Totally disagree, Can't do it at all  2-Hardly true, A little agree, Can do it occasionally  3-Moderately true, Majority agree, Most can do  4-Exactly true, Totally agree, Can always be done |
| 2. Even if someone opposes me, I can find the means and ways  to get what I want (Even if others don't agree with you, like your actions or ideas, you can still get what you want and achieve the final goal) |  |
| 3.... |  |
| 4. ... |  |
| 5. ... |  |
| 6. ... |  |
| 7. ... |  |
| 8. ... |  |
| 9. ... |  |
| 10. I can usually handle whatever comes my way (No matter what problem you encounter, you can easily solve it) |  |

1. **The 14-item resilience scale, RS-14**

**Overview:**

Please read the 14 sentences below. The numbers 1-7 indicate your level of agreement with the statement.

For example, if you completely disagree with a statement, choose "1"; if you completely agree with the statement, choose "7"; if you remain neutral with this statement, choose "4".

| items |  |
| --- | --- |
| 1. I usually manage one way or another (You usually have many ways to handle things ) | 1-Strongly disagree, Can't do it at all  2-Disagree  3-Slightly disagree  4-neutral, neither agree nor disagree  5-Slightly agree  6-Agree  7-Strongly agree, Can always be done |
| 2. I feel proud that I have accomplished things in life (You are proud of what you have achieved so far) |  |
| 3. ... |  |
| 4. ... |  |
| 5. ... |  |
| 6. ... |  |
| 7. ... |  |
| 8. ... |  |
| 9. ... |  |
| 10. ... |  |
| 11. ... |  |
| 12. ... |  |
| 13. ... |  |
| 14. When I'm in a difficult situation, I can usually find my way out of it (When you encounter troubles, you can find a way out of it) |  |
